# Photon-counting detector CT and energy-integrating detector CT for trabecular bone microstructure analysis of cubic specimens from human radius

**DOI:** 10.1186/s41747-022-00286-w

**Published:** 2022-07-27

**Authors:** Benjamin Klintström, Lilian Henriksson, Rodrigo Moreno, Alexandr Malusek, Örjan Smedby, Mischa Woisetschläger, Eva Klintström

**Affiliations:** 1 Department of Biomedical Engineering and Health Systems, KTH Royal Institute of Technology, Hälsovägen 11C, SE-14157 Huddinge, Sweden; 2 Center for Medical Image Science and Visualization (CMIV), Linköping University, SE-58185 Linköping, Sweden; 3 Department of Radiology and Department of Health, Medicine and Caring Sciences, Linköping University, SE-58185 Linköping, Sweden; 4 Radiation Physics, Department of Health, Medicine and Caring Sciences, Linköping University, SE-58183 Linköping, Sweden

**Keywords:** Cancellous bone, Osteoporosis, Radius, Tomography (x-ray computed), X-ray microtomography

## Abstract

**Background:**

As bone microstructure is known to impact bone strength, the aim of this *in vitro* study was to evaluate if the emerging photon-counting detector computed tomography (PCD-CT) technique may be used for measurements of trabecular bone structures like thickness, separation, nodes, spacing and bone volume fraction.

**Methods:**

Fourteen cubic sections of human radius were scanned with two multislice CT devices, one PCD-CT and one energy-integrating detector CT (EID-CT), using micro-CT as a reference standard. The protocols for PCD-CT and EID-CT were those recommended for inner- and middle-ear structures, although at higher mAs values: PCD-CT at 450 mAs and EID-CT at 600 (dose equivalent to PCD-CT) and 1000 mAs. Average measurements of the five bone parameters as well as dispersion measurements of thickness, separation and spacing were calculated using a three-dimensional automated region growing (ARG) algorithm. Spearman correlations with micro-CT were computed.

**Results:**

Correlations with micro-CT, for PCD-CT and EID-CT, ranged from 0.64 to 0.98 for all parameters except for dispersion of thickness, which did not show a significant correlation (*p* = 0.078 to 0.892). PCD-CT had seven of the eight parameters with correlations *ρ* > 0.7 and three *ρ* > 0.9. The dose-equivalent EID-CT instead had four parameters with correlations *ρ* > 0.7 and only one *ρ* > 0.9.

**Conclusions:**

In this *in vitro* study of radius specimens, strong correlations were found between trabecular bone structure parameters computed from PCD-CT data when compared to micro-CT. This suggests that PCD-CT might be useful for analysing bone microstructure in the peripheral human skeleton.

## Key points

- Photon-counting detector CT (PCD-CT) showed strong correlations with micro-CT for bone structure parameters.
- PCD-CT showed less over-/underestimation of bone structure parameters when compared to energy-integrating detector CT (EID-CT).
- PCD-CT could enable the use of a lower radiation dose at the same or better true resolution when compared to EID-CT.

## Background

Osteoporosis is a skeletal disease where multiple pathogenic processes lead to loss of bone mass and changes in the microarchitecture [1]. Both mineral content and the internal bone microstructure have an impact on bone strength [2, 3]. Osteoporotic bone has become more fragile with an increased risk of fractures. Osteoporotic fractures of central body parts like the hip and spine are associated with high mortality and morbidity, representing a high economic burden for society [4, 5]. Sarcopenia, another typical feature of ageing, is also associated with falls and fractures causing high morbidity [6]. Determining the microarchitecture of bone, both cortical and trabecular, is essential for an accurate assessment of bone strength [7]. Bone structure can be visualised using three-dimensional (3D) computed tomography (CT) variants like micro-computed tomography (micro-CT), high-resolution peripheral quantitative computed tomography (HR-pQCT), dental cone beam computed tomography (dCBCT), multislice energy-integrating detector CT (EID-CT) and multislice photon-counting detector CT (PCD-CT) with energy discrimination capability [8–14]. Trabecular and cortical bone structure parameters can be described using a nomenclature standardised according to Parfitt et al. [9].

Parameters computed from micro-CT are usually used as a standard reference for the analysis of bone microarchitecture [8]. Due to the small imaging volume, high radiation dose and long imaging time inherent in these scans, micro-CT is only feasible for bone specimens and small, sedated research animals. HR-pQCT can be used for *in vivo* analysis of bone microstructure in the extremities [10, 15]. Previous studies using HR-pQCT data showed that deficits in trabecular and cortical bone density and structure are independent factors when assessing the future fracture risk in older women and men [16]. Besides HR-pQCT, dCBCT has shown promising results in this field [11, 12]. In addition, dCBCT can be used to scan the jaw and, with modifications, the extremities [17].

EID-CT is used to examine osteoporotic fractures of central body parts like the hip and spine [13]. EID-CT scanners have detector pixels that range in size from 0.5 to 0.625 mm at the isocentre, resulting in a low spatial resolution, making the analysis of bone microstructure difficult. Bone microstructure analyses based on EID-CT data may therefore result in severe overestimations of trabecular bone structure parameters [18]. Moreover, EID-CT has shown weaker correlations with micro-CT than HR-pQCT and dCBCT in regard to certain bone structure parameters like *trabecular thickness* (Tb.Th) and *bone volume over total volume* (BVTV) [12, 19, 20].

PCD-CT is a new technology that uses small detector elements registering individual photon interactions. The benefits of PCD-CT are higher spatial resolution and better contrast [21, 22]. Compared to EID-CT, it better suppresses electronic noise and improves the imaging of small structures [23, 24]. The PCD-CT used in this study had a detector pixel size of 0.25 mm at the isocentre; the same detector and geometry were used in reference [25]. A larger reconstruction matrix is typically used to utilise the higher spatial resolution of PCD-CT when compared to EID-CT. Its use has been shown to improve the assessment of small structures related to lung disease and affects the analysis of bone microstructures favourably [26, 27]. Given the importance of bone microstructure for overall bone strength, one important remaining question is whether the increased resolution and decreased noise of PCD-CT is adequate to allow such an analysis.

The aim of this *in vitro* study is, therefore, to evaluate if the emerging PCD-CT technique may be used for measurements of trabecular bone structure by scanning cubic sections of human cadaveric wrist specimens in both one EID-CT and one PCD-CT. Images provided by the two scanners were quantitatively compared; the focus was on structure parameters known to influence bone strength. Micro-CT was used as a reference method.

## Methods

### Material

The specimens used in this *in vitro* study consisted of 14 nearly cubic bone pieces from cadaveric human wrists donated for medical research at the University of California in compliance with the prevailing ethical guidelines. All specimens have been used in previous studies [11, 20]. The specimens had sides of 12−15 mm, at least one of which consisted of cortical bone. The specimens were chemically defatted and stored in individual tap water-filled test tubes at room temperature. Multiple repeated scans in a dCBCT (Accuitomo 80, J. Morita Mfg. Corp., Kyoto, Japan) with the same examination protocol and method for analysis were made multiple years apart to test the durability of the specimens and the reproducibility of the analysis [28]. During scanning, the test tubes were placed inside a paraffin cylinder with a diameter of 100 mm to mimic the forearm with soft tissue surrounding the bone.

### Scanning protocols

Scanning protocols used on both the PCD-CT and EID-CT scanners were the ones recommended by the manufacturer for imaging the inner- and middle-ear bone structures, although using higher tube current-time product (mAs) settings. For the PCD-CT, the highest mAs available was used. For the EID-CT, both the highest available mAs and the mAs that achieved the same CTDI_vol_ as the PCD-CT were used. Details of the scan and reconstruction parameters can be seen in Table 1.

**Table 1:** Scanning and reconstruction parameters for multislice computed tomography using energy-integrating detectors (EID-CT) and multislice computed tomography using photon-counting detectors (PCD-CT)

Scanner	Tube voltage (kVp)	Quality reference tube load (mAs)	Effective tube load (mAs)	Pitch	CTDI_vol_ (mGy)	Field of view (mm)	Matrix size	Intra-slice voxel size (μm)	Slice increment (μm)	Slice thickness (μm)	Voxel size (μm)	Kernel
PCD-CT 51	120	450	~170	0.6	29	51	1,024	50	50	200	50	Qr80u
PCD-CT 30	120	450	~170	0.6	29	30	2,048	15	50	200	50	Qr80u
PCD-CT 18	120	450	~170	0.6	29	18	2,048	8.8	50	200	50	Qr80u
EID-CT 1000	120	1,000	~360	0.6	49	51	512	100	100	400	100	Ur81-3
EID-CT 600	120	600	~210	0.6	29	51	512	100	100	400	100	Ur77-3

The computed tomography dose index (CTDI_vol_) was supplied by the CT scanner software. The numbers after the type of scanner correspond to the field of view in mm (PCD-CT) and quality reference tube load in mAs (EID-CT)

The PCD-CT scanner used in this study was a research prototype SOMATOM Count Plus (Siemens Healthineers, Erlangen, Germany). Each specimen was reconstructed with three different combinations of field of view (FOV) and matrix size, which resulted in an intra-slice pixel size of 50 μm, 15 μm and 8.8 μm, respectively. The slice width was 200 μm, and the slice increment was 50 μm.

The EID-CT scans were acquired using a SOMATOM Force scanner (Siemens Healthineers, Erlangen, Germany) using the InnerEar UHR protocol. EID-CT data sets were reconstructed with a matrix of 512 × 512, FOV of 51 mm, slice width of 400 μm and slice increment of 100 μm with three different kernels, UR73 Admire 3, UR77 Admire 3 and UR81 Admire 3.

Data acquired with a SkyScan 1,176 micro-CT scanner (Bruker micro-CT, Kontich, Belgium) were used as reference. The scanning parameters were tube voltage of 65 kVp, tube current of 385 μA and 1-mm aluminium filter for beam hardening. The FOV was adapted to every specimen, and the acquisition time was approximately 2 h per sample.

### Data processing, segmentation and analysis of structure parameters

The PCD-CT data at 15 and 8.8 μm were downsampled to a voxel size of 50 μm (matching the slice increment) using cubic interpolation to achieve isotropic voxels. The PCD-CT and EID-CT volumes were manually registered to the micro-CT volumes in a two-step process using the registration manual module in MeVisLab (MeVis Medical Solutions AG, Bremen, Germany) to identify the same volumes of interest for the different scanners. For segmentation of the PCD-CT and EID-CT data, an in-house developed implementation of the automated region growing (ARG) algorithm that requires no manual intervention was used, as described in previous publications [29, 30]. The micro-CT data were segmented using Otsu thresholding [31]. Histograms of the scaled intensity for the final segmentation of the same specimen were calculated for each modality in Matlab version R2020a Update 3 (Mathworks, Portola Valley, USA) after normalising the mean and standard deviation of the segmented background (mean of 0 and standard deviation of 500).

The following bone structure parameters were calculated in 3D for each volume from the binary, segmented data:

- *Bone volume over total volume* (BVTV): the fraction (%) of the total number of voxels in the analysed volume segmented as bone
- *Trabecular thickness* (Tb.Th): the width (mm) of the trabeculae; it was calculated as described in [32].
- *Trabecular separation* (Tb.Sp): the minimum distance (mm) between the edges of neighbouring trabeculae; it is calculated using the same method as Tb. Th on the inverted segmentation masks.
- *Trabecular spacing* (Tb.Sc): the minimum distance (mm) between the midlines of neighbouring trabeculae; it was calculated using the same method as for Tb. Sp but with the inverted skeleton of the bone (centre of the bone structures) as the input instead of the segmented bone (we computed Tb.Sc instead of the more commonly used trabecular number (Tb.N), which is the reciprocal of Tb.Sc, because unlike Tb.N, Tb.Sc shares the same unit with Tb.Th and Tb.Sp, which eases the comparisons).
- *Trabecular nodes* (Tb.Nd): the number of voxels classified as a node in the trabecular network divided by the volume of the analysed volume in cubic millimetres (mm^3^). The nodes are defined as intersections in the skeleton of the bone.

BVTV and Tb.Nd are only defined as a single measure for the entire volume. On the other hand, the method used for calculating Tb.Th, Tb.Sp and Tb.Sc creates 3D local maps, and they therefore vary within each individual volume. The convention is to only present the mean for the entire volume (over all voxels). To better represent these parameters, we have chosen to also present a measure of the dispersion within each volume called s(Tb.Th), s(Tb.Sp) and s(Tb.Sc). These are the standard deviations of the 3D local map for each of the three parameters.

Furthermore, we also used the *contrast-to-noise* (CNR) ratio (unitless) to compare the different modalities. For each bone sample, the CNR was calculated as the difference in mean intensity between foreground and background divided by the standard deviation of the background. The skeletonised representation of the bone was used as the foreground signal, and the skeletonised representation of the water was used as the background signal.

In summary, we computed BVTV, Tb.Th, s(Tb.Th), Tb.Sp, s(Tb.Sp), Tb.Sc, s(Tb.Sc), Tb.Nd and CNR.

### Statistical analysis

To test for normality, we used the Shapiro-Wilk test in *R* version 4.0.2 (R Foundation, Indianapolis, USA). Spearman rank correlations with *p*-values for PCD-CT *versus* micro-CT and EID-CT *versus* micro-CT were calculated using *corr* in Matlab version R2020a Update 3 (Mathworks, Portola Valley, USA). Median, 1st and 3rd quartile were calculated for each modality and structural parameter.

### Visual presentation

Slices along the axial (*xy*) and coronal (*xz*) plane were extracted for one specimen from each modality after registration in MeVisLab (MeVis Medical Solutions AG, Bremen, Germany). For the same specimen, the 3D local maps for Tb.Th were extracted from the Matlab code and colour graded with the minimum value represented as blue, maximum as red and the midpoint between maximum and minimum as green.

## Results

### Test for normality and descriptive statistics

Normality testing of the analysed structure parameters resulted in the rejection of the normality hypothesis (*p* < 0.05) for s(Tb.Sc) (*p* = 0.003–0.023) and s(Tb.Sp) (*p* = 0.009–0.017) for the EID-CT and PCD-CT. For micro-CT, it resulted in *p* < 0.05 for the same parameters and also for Tb.Sc and Tb.Sp (*p* = 0.046, 0.029, 0.015, and 0.013, respectively).

Both the PCD-CT and EID-CT overestimated BVTV compared to micro-CT. The median of the EID-CT was 3.8 to 3.9 times that of micro-CT, and for PCD-CT, the same values were 3.6 to 3.7 (Table 2). The same could be seen for Tb.Th, where the EID-CT overestimated the parameter by a factor of 3.6 to 3.8 and the PCD-CT by a factor of 3.2. On the other hand, Tb.Nd was underestimated by both EID-CT and PCD-CT; EID-CT underestimated it by a factor of 10.8 to 11.9 and PCD-CT by a factor of 6.7 to 6.9. The other structural parameters showed values that were almost in the same range as the micro-CT.

**Table 2:** Descriptive statistics for the eight structure parameters and CNR

Scanner	Average measures						Dispersion measures		
	BVTV	Tb.Th	Tb.Sc	Tb.Sp	Tb.Nd	CNR	s(Tb.Th)	s(Tb.Sc)	s(Tb.Sp)
PCD-CT^2^ 51	0.32	0.45	1.16	0.79	1.36	8.07	0.15	0.46	0.37
	(0.27; 0.36)	(0.43; 0.46)	(1.08; 1.30)	(0.68; 0.88)	(1.06; 1.71)	(6.89; 9.56)	(0.14; 0.16)	(0.39; 0.54)	(0.31; 0.45)
PCD-CT^2^ 30	0.32	0.44	1.16	0.79	1.39	7.73	0.15	0.46	0.37
	(0.27; 0.36)	(0.43; 0.45)	(1.07; 1.30)	(0.68; 0.88)	(1.10; 1.77)	(6.55; 9.31)	(0.14; 0.16)	(0.40; 0.54)	(0.30; 0.46)
PCD-CT^2^ 18	0.33	0.45	1.15	0.78	1.39	7.94	0.15	0.46	0.36
	(0.28; 0.37)	(0.43; 0.46)	(1.06; 1.29)	(0.67; 0.87)	(1.08; 1.76)	(6.44; 9.16)	(0.15; 0.16)	(0.38; 0.53)	(0.30; 0.46)
EID-CT^1^ 1000	0.34	0.50	1.20	0.85	0.86	6.45	0.13	0.44	0.35
	(0.28; 0.37)	(0.48; 0.50)	(1.12; 1.34)	(0.75; 0.94)	(0.64; 1.07)	(5.34; 7.05)	(0.12; 0.14)	(0.38; 0.52)	(0.31; 0.44)
EID-CT^1^ 600	0.35	0.53	1.23	0.85	0.78	5.93	0.15	0.45	0.35
	(0.30; 0.39)	(0.52; 0.54)	(0.16; 0.36)	(0.76; 0.94)	(0.60; 0.97)	(4.95; 6.39)	(0.14; 0.16)	(0.40; 0.51)	(0.32; 0.46)
Micro-CT	0.09	0.14	1.07	0.91	9.32	24.94	0.06	0.30	0.27
	(0.07; 0.12)	(0.13; 0.16)	(0.86; 1.17)	(0.73; 1.04)	(6.78; 11.04)	(21.44; 26.50)	(0.05; 0.06)	(0.24; 0.36)	(0.22; 0.32)

Data are presented as median (1st quartile, 3rd quartile)

*BVTV* Bone volume fraction, *CNR* Contrast-to-noise ratio, *EID-CT* Multislice computed tomography using energy-integrating detectors, *Micro-CT* Micro-computed tomography, *PCD-CT* Multislice computed tomography using photon-counting detectors, *Tb.Nd* Trabecular nodes, *Tb.Th* Trabecular thickness, *Tb.Sc* Trabecular spacing, *Tb.Sp* Trabecular separation. s(Tb.Th), s(Tb.Sc) and s(Tb.Sp) are the intra-volume standard deviation for Tb.Th, Tb.Sc and Tb.Sp, respectively. The numbers after the type of scanner correspond to the field of view in mm (PCD-CT) and quality reference tube load in mAs (EID-CT)

The highest CNR was observed for the micro-CT at 25 followed by the PCD-CT at 7.7 to 8.1. The EID-CT had a CNR at 5.9 to 6.5. For the PCD-CT, the combination of a FOV of 51 mm and a matrix size of 1,024 × 1,024 (PCD-CT 51) showed the highest CNR (see Table 2).

### Correlations

**Fig. 1:**
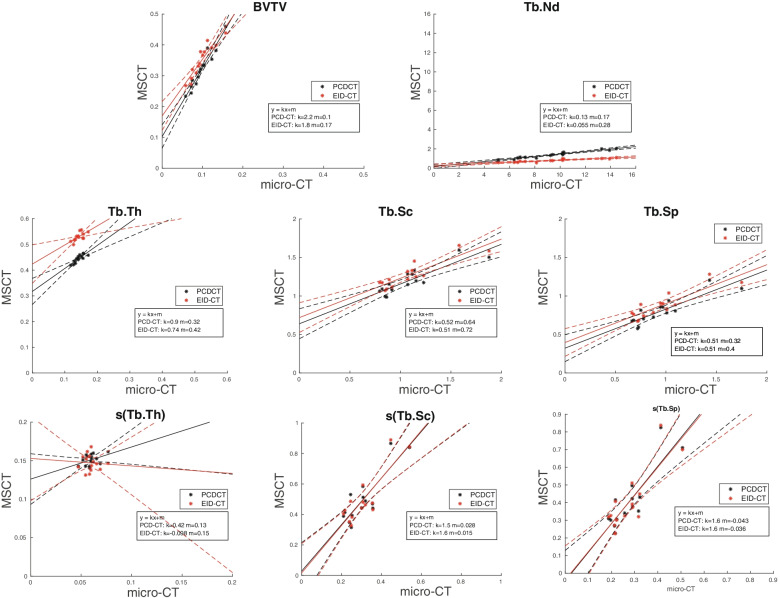
Scatterplots of bone structure parameters. *For each specimen, solid line for the fitted linear model, dashed line for the 95% confidence intervals of the fitted linear model. Multislice computed tomography using energy-integrating detectors (EID-CT) is coloured red, while multislice computed tomography using photon-counting detectors (PCD-CT) is coloured black. For the PCD-CT, the combination of a field of view of 51 mm and a matrix of 1024 × 1024 was chosen and for the EID-CT the mAs setting with the same CTDI_vol_ as the PCD-CT was used. *BVTV* Bone volume fraction, *Micro-CT* Micro-computed tomography, *Tb.Th* Trabecular thickness, *Tb.Nd* Trabecular nodes, *Tb.Sc* Trabecular spacing, *Tb.Sp* Trabecular separation. Parameter for the average of each specimen and s(parameter) for the dispersion measure

Scatterplots for each structure parameter are shown in Fig. 1, and correlation coefficients (*ρ*) are given in Table 3. The PCD-CT showed correlation (*ρ*) with micro-CT for trabecular bone structure parameters between 0.44 and 0.98 The lowest correlation was found for s(Tb.Th) at *ρ* = 0.44, which was not statistically significant (*p* = 0.116). The highest correlation was found for BVTV followed by Tb.Nd; both showed correlations with micro-CT *ρ* ≥ 0.95. Changing the combination of FOV and matrix size resulted in minor differences in correlations. The EID-CT had correlations that ranged from *ρ* = -0.04 to *ρ* = 0.98. At the higher radiation dose, the EID-CT showed higher correlations (*ρ*) to micro-CT using the Ur81 kernel, while Ur77 showed higher correlations at the lower radiation dose protocol. Thus, for each dose level, data for the kernel yielding the highest correlation values are given in Table 3.

**Table 3:** Spearman rank correlation with micro-CT: coefficients with 95% confidence intervals for the eight analysed structure parameters

Scanner	Average measures					Dispersion measures		
	BVTV	Tb.Th	Tb.Sc	Tb.Sp	Tb.Nd	s(Tb.Th)	s(Tb.Sc)	s(Tb.Sp)
PCD-CT^2^ 51	**0.98 **	0.92	0.83	0.84	0.95	0.44	0.67	**0.81 **
	**(0.94; 0.99)**	(0.75; 0.97)	(0.53; 0.94)	(0.56; 0.95)	(0.84; 0.98)	(−0.12; 0.79)	(0.22; 0.89)	**(0.48; 0.94)**
	***p*< 0.001**	*p* < 0.001	*p* < 0.001	*p* < 0.001	*p* < 0.001	*p* = 0.116	*p* = 0.010	***p*= 0.001**
PCD-CT^2^ 30	**0.98 **	**0.93 **	**0.85 **	**0.87 **	**0.96 **	**0.49 **	**0.71 **	**0.81 **
	**(0.94; 0.99)**	**(0.79; 0.98)**	**(0.59; 0.95)**	**(0.64; 0.96)**	**(0.89; 0.99)**	**(−0.05; 0.81)**	**(0.30; 0.90)**	**(0.48; 0.94)**
	***p*< 0.001**	***p*< 0.001**	***p*< 0.001**	***p*< 0.001**	***p*< 0.001**	***p*= 0.078**	***p*= 0.006**	***p*= 0.001**
PCD-CT^2^ 18	**0.98 **	0.92	0.83	**0.87 **	0.95	0.46	0.71	**0.81 **
	**(0.94; 0.99)**	(0.75; 0.97)	(0.54; 0.95)	**(0.64; 0.96)**	(0.85; 0.98)	(−0.09; 0.80)	(0.29; 0.90)	**(0.48; 0.94)**
	***p*< 0.001**	*p* < 0.001	*p* < 0.001	***p*< 0.001**	*p* < 0.001	*p* = 0.097	*p* = 0.006	***p*= 0.001**
EID-CT^1^ 1000	**0.98 **	0.77	0.81	0.77	0.95	−0.04	0.69	0.76
	**(0.94; 0.99)**	(0.40; 0.92)	(0.49; 0.94)	(0.40; 0.92)	(0.85; 0.98)	(−0.56; 0.50)	(0.25; 0.89)	(0.38; 0.92)
	***p*< 0.001**	*p* = 0.001	*p* = 0.001	*p* = 0.002	*p* < 0.001	*p* = 0.892	*p* = 0.008	*p* = 0.002
EID-CT^1^ 600	0.97	0.64	0.83	0.80	0.88	−0.04	0.66	0.70
	(0.90; 0.99)	(0.17; 0.88)	(0.54; 0.95)	(0.46; 0.93)	(0.66; 0.96)	(−0.56; 0.50)	(0.19; 0.88)	(0.26; 0.90)
	*p* < 0.001	*p* = 0.015	*p* < 0.001	*p* < 0.001	*p* < 0.001	*p* = 0.892	*p* = 0.013	*p* = 0.007

Bold numbers indicate the highest correlation for each parameter. *BVTV* Bone volume fraction, *EID-CT* Multislice computed tomography using energy-integrating detectors (^1^), *PCD-CT* Multislice computed tomography using photon-counting detectors (^2^), *Tb.Nd* Trabecular nodes, *Tb.Th* Trabecular thickness, *Tb.Sc* Trabecular spacing, *Tb.Sp* Trabecular separation. s(Tb.Th), s(Tb.Sc) and s(Tb.Sp) are the intra-volume standard deviation for Tb.Th, Tb.Sc and Tb.Sp, respectively. The numbers after the type of scanner correspond to the field of view in mm (PCD-CT) and quality reference tube load in mAs (EID-CT)

### Visual presentation

**Fig. 2:**
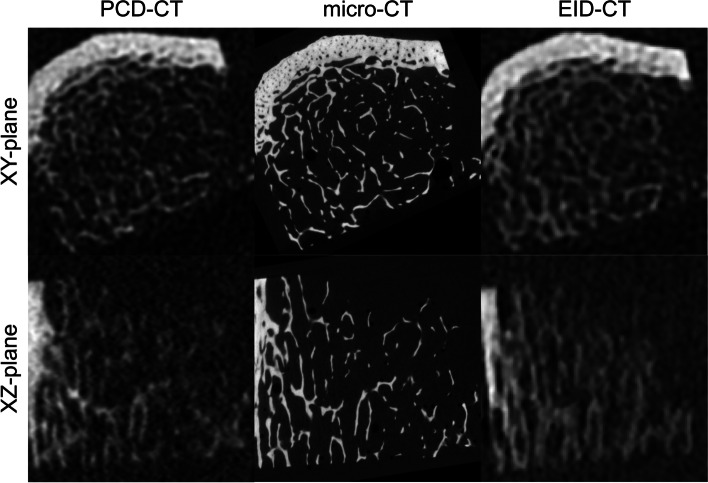
Slices from the different devices used in the study in the *xy*-plane and *xz*-plane (*x* horizontally in both planes). For the multislice computed tomography using photon-counting detectors (PCD-CT), the combination of a field of view of 51 mm and a matrix of 1,024 × 1,024 was chosen and for the multislice computed tomography using energy-integrating detectors (EID-CT) the mAs setting with the same CTDI_vol_ as the PCD-CT was used. The PCD-CT and EID-CT slices are presented as they were scanned without any rotation applied, while the micro-CT has been registered to the PCD-CT volume. *Micro-CT* Micro-computed tomography

As seen in Fig. 2, the PCD-CT scans show less defined edges in the *z* direction when compared to the *x* or *y* direction. One can also see in the same figure that the difference is even more pronounced in the EID-CT.

**Fig. 3:**
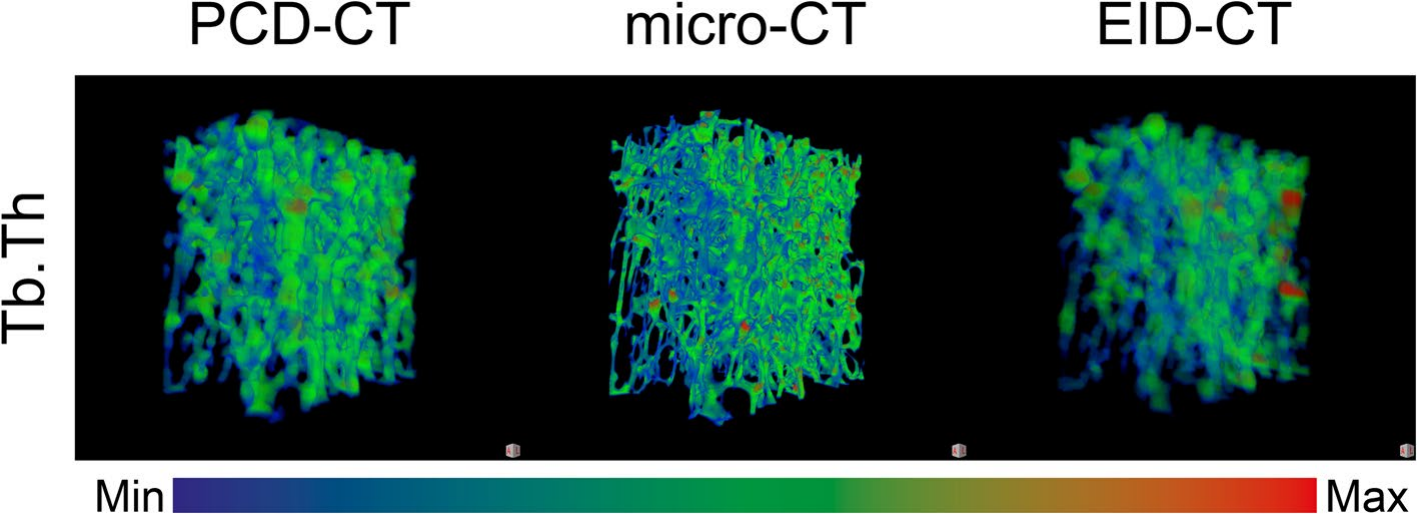
Three-dimensional map of trabecular thickness (Tb.Th) within one of the analysed specimens. For the multislice computed tomography using photon-counting detectors (PCD-CT), the combination of a field of view of 51 mm and a matrix of 1,024 × 1,024 was chosen and for the multislice computed tomography using energy-integrating detectors (EID-CT) the mAs setting with the same CTDIvol as the PCD-CT was used. The colour scale was adjusted so that the minimum and maximum values for each modality were represented by pure blue and red, respectively, with the midpoint between them being represented by pure green. *Micro-CT* Micro-computed tomography

As shown in Fig. 3, the thickness maps are consistent between the modalities; areas of high thickness for one modality correspond to areas of high thickness for the other modalities.

### Contrast and segmentation evaluation

**Fig. 4:**
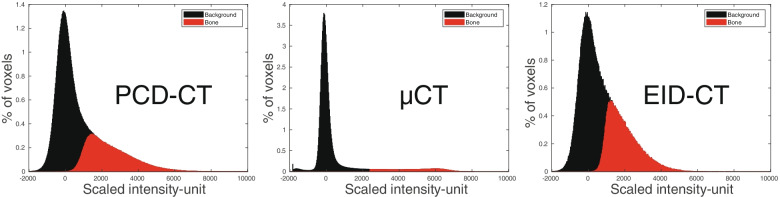
Stacked histogram of scaled intensity for the voxels segmented as bone and background for one of the bone specimens. For multislice computed tomography using photon-counting detectors (PCD-CT) and multislice computed tomography using energy-integrating detectors (EID-CT) data, the segmentation was achieved by the automated region growing algorithm, while the micro-CT data was segmented using Otsu thresholding. For the PCD-CT, the combination of a field of view of 51 mm and a matrix of 1,024 × 1,024 was chosen and for the EID-CT the mAs setting with the same CTDIvol as the PCD-CT was used. *Micro-CT* Micro-computed tomography

Stacked histograms of the scaled intensity after segmentation to bone and background are presented in Fig. 4, where the EID-CT and PCD-CT data were segmented using the ARG algorithm, while micro-CT data were segmented using Otsu thresholding. The micro-CT demonstrates a clear separation between bone and background with two distinct distributions. EID-CT and PCD-CT, on the other hand, show distributions that are not clearly separated. Interestingly, one can see that the ARG algorithm yields a smooth transition between the background and bone distributions over a range of intensity values.

## Discussion

In this *in vitro* study, PCD-CT produced results that make the technique potentially promising for imaging and analysing bone microstructure. Regardless of the used reconstruction parameters, correlations with micro-CT showed *ρ* ≥ 0.7 for six of the eight measured bone structure parameters indicating a high correlation [33]. In turn, at the same radiation dose, the EID-CT had only four parameters with correlations showing *ρ* ≥ 0.7. The PCD-CT had three correlations *ρ* ≥ 0.9, indicating a very high correlation, while the EID-CT with equivalent dose had a correlation *ρ* ≥ 0.9 only for BVTV. The highest correlations with micro-CT were found for FOV 30 mm and matrix 2,048 × 2,048, where all but one parameter, s(Tb.Th), had correlations *ρ* ≥ 0.7. PCD-CT tends to overestimate the amount of bone and trabecular thickness and underestimate the number of intersections of the trabecular network (Tb.Nd) (see Table 2), however, to a lesser extent than the EID-CT. Also, compared to other clinically available devices like dCBCT and HR-pQCT, PCD-CT performs well for microstructure analysis when used in conjunction with our segmentation method [28].

At the moment, imaging of the distal tibia and radius using HR-pQCT devices is the most validated clinically available method for *in vivo* analysis of trabecular microstructure [34, 35]. The correlation with micro-CT as well as amount of over-/underestimation varies depending on the bone structure parameter analysed [36]. One drawback of HR-pQCT, however, is the low number of devices available for clinical use. A device type that is more widely spread and has shown promise for the analysis of trabecular microstructure is dCBCT, which can be used to scan the jaw and, with special adaptations, the wrist [17, 37]. The actual resolution and reconstructed voxel size is about the same for HR-pQCT and dCBCT, and they have shown similar correlations with micro-CT [28]. Imaging of the hip or spine is, however, not possible with either of these techniques. For EID-CT units, the detector pixel sizes are about 0.5 to 0.625 mm at the isocentre, limiting their resolution.

The PCD-CT used in this study has smaller pixels, at 0.25 mm at the isocentre, enabling a higher actual resolution. Previous studies have shown that the average trabecular thickness is about 100 μm in humans [38]. The differences observed in this study are therefore mostly likely partly explained by the smaller pixels of the PCD-CT, combined with PCD-CT’s inherent ability to suppress electronic noise. However, the slice thickness of 200 μm will still result in voxels that consist of a mix of bone and background, due to the partial volume effect, which might affect the ability to analyse trabecular bone microstructure. The increment of 50 μm used in this study could have counteracted this to some degree, but the resolution would still have been limited by the slice thickness (Fig. 2). It was not possible to scan and reconstruct at a smaller slice thickness than 200 μm with the prototype PCD-CT used in this study. Since whole-body PCD-CT is still under development, we expect that in the future, one could possibly decrease the slice thickness even further, hopefully to the same size or smaller than the thickness of trabeculae.

There are certain limitations to this study. First, the number of bone specimens are rather limited. However, these specimens have been scanned and analysed many times, both repeatedly in the same dCBCT with the same protocol to verify reproducibility and in multiple different devices using different protocols and segmentation methods. This consistent approach enables comparisons with earlier published results [11, 20, 28, 39]. The PCD-CT and EID-CT protocols used here were those recommended by the vendor for imaging of inner- and middle-ear structures changed to use the highest possible tube current-time product (mAs) available. Earlier studies of the tiny bone structures in the inner ear have shown the possibility of using a lower radiation dose with PCD-CT compared with EID-CT while preserving the image quality [24, 40]. In this study, we found that even at a higher radiation dose, the EID-CT could not match the performance of the PCD-CT. We plan to further study the impact of radiation dose and other imaging parameters on the analysis of bone microstructure using PCD-CT before fully assessing its potential for *in vivo* imaging.

If the results of this study and the strong correlations found for PCD-CT with micro-CT are replicated by future *in vivo* studies, this might enable analysis of bone microstructure in a clinical workflow. Since multislice CT is used in the clinical workflow of fractures, this could enable opportunistic screening for pathological bone microstructure changes [41, 42]. Since PCD-CT is capable of energy resolution (*i.e.,* the ability to measure the energy of each detected x-ray photon [22]), this could enable combined bone density and bone microstructure analysis potentially revolutionising diagnosis and management of osteoporosis.

In conclusion, strong correlations were found between trabecular bone structure parameters computed from PCD-CT data and micro-CT under simulated *in vivo* conditions. This suggests that PCD-CT might be useful for analysing bone microstructure in the peripheral human skeleton.

## Data Availability

The datasets used and/or analysed during the current study are available from the corresponding author on reasonable request.

## Abbreviations

3D: Three dimensional
ARG: Automated region growing
BVTV: Bone volume over total volume
CNR: Contrast-to-noise ratio
CT: Computed tomography
CTDI_vol_: CT dose index volume
dCBCT: Dental cone beam CT
EID-CT: Energy-integrating detector CT
FOV: Field of view
HR-pQCT: High-resolution peripheral quantitative CT
PCD-CT: Photon-counting detector CT
Tb.N: Trabecular number
Tb.Nd: Trabecular nodes
Tb.Sc: Trabecular spacing
Tb.Sp: Trabecular separation
Tb.Th: Trabecular thickness
